# The Time of Diagnosis and Surgical Treatment of Congenital Cryptorchidism: A Single Center’s Observational Study in Greece

**DOI:** 10.7759/cureus.51580

**Published:** 2024-01-03

**Authors:** Christos Kaselas, Maria Florou, Maria Tirta, Sophia Bitzika, Daphne Sidiropoulou, Ioannis Spyridakis

**Affiliations:** 1 2nd Department of Pediatric Surgery, Aristotle University of Thessaloniki, Papageorgiou General Hospital, Thessaloniki, GRC; 2 School of Medicine, Aristotle University of Thessaloniki, Thessaloniki, GRC

**Keywords:** orchidopexy, diagnosis, guidelines, cryptorchidism, children

## Abstract

Purpose: Congenital cryptorchidism or undescended testes (UDT) is one of the most common congenital abnormalities in newborns. Current guidelines recommend that surgical management should be scheduled by the 12th month and no later than the 18th month of the child’s life. This is the first study to evaluate the age of diagnosis and surgical treatment of children with UDT in Greece, as well as the compliance with current guidelines worldwide.

Methods: A retrospective analysis of patients with UDT who underwent orchidopexy from 2015 to 2019 was conducted. Patient age at diagnosis and orchidopexy and the meantime between were recorded. Patients were separated into groups, based on the diagnosis age: group A, diagnosis until the 11th month; group B, diagnosis between the 12th and 18th month; and group C, diagnosis at >18th month.

Results: We identified 217 children who were diagnosed with UDT and underwent orchidopexy in our department. The majority of the patients (47.4%) had right-sided UDT, while 25.3% of them had UDT on both sides. There were 89 (41%) children in group A, 20 (9.2%) in group B, and 108 (49.8%) males in group C. The median age at diagnosis was 18 months (range: 1-164 months), while for groups A, B, and C, the median age at diagnosis was five, 15, and 71.5 months, respectively. The median age at orchidopexy was 23 months (range: 6-166 months), and for each aforementioned group, it was 11, 16.5, and 74 months. The median waiting time for the orchidopexy was 84 days (range: 1-692 days), and for each group, it was 157, 42, and 56 days, respectively. The delay between diagnosis and surgery was significantly greater for group A compared to groups B and C (p _A versus B_ = 0.01 and p _A versus C_< 0.0001), while there was no difference in the delay between groups B and C (p > 0.05).

Conclusions: Patient age at diagnosis and applied orchidopexy was within the recommended range for almost half of the patients. The rest of them had delayed diagnosis and surgery due to delayed referral. In delayed cases, the time from diagnosis to treatment was significantly shorter. Early surgical referral leading to prompt treatment will increase compliance with the guidelines and improve the quality and the outcomes of the provided health-care services.

## Introduction

Congenital cryptorchidism or undescended testis (UDT) occurs in 3%-5% of full-term neonates, but rates are as high as 45% in preterm neonates with low birth weight. It is considered one of the most common congenital abnormalities of the genitourinary tract in newborn males [1,2]. Cryptorchidism rates drop to 0.8%-1% in one-year-old infants, due to a spontaneous descent of the testicles that can take place from the third to the sixth month of life (corrected gestational age). During this period called mini-puberty, there is a transient increase in gonadotropins and androgens, which contributes to the spontaneous movement of the testes down to the scrotum [3,4]. The testicles that have not descended into the scrotum by the sixth month of life (corrected gestational age) are considered unlikely to descend later, and so, the sixth month is considered the upper time limit for the diagnosis of congenital cryptorchidism [5-8]. The latest guidance from several scientific associations demonstrates that the diagnosis of infants with congenital cryptorchidism should be made from the third to the sixth month, in order to schedule the surgical treatment by the 12th month and no later than the 18th month of the child’s life [9-12].

Suboptimally, compliance with these guidelines remains poor, and a considerable number of orchidopexies continue to be performed beyond one year of age [13]. Specifically, the mean age of children undergoing orchidopexy has a wide distribution in childhood and adolescence, with two peaks occurring in the second year and the 10th-11th year of the child’s life [14]. Obviously, many males do not receive diagnosis and/or treatment for UDT at the optimal age. The adherence to the guidelines, the prevalence of the delayed orchidopexy, and the factors contributing to such delays have been poorly examined in the literature. Even more, the age at UDT diagnosis and/or orchidopexy could be indicators of the promptitude and the quality of health-care services in a pediatric surgical department [15].

Thus, we investigated the treatment pathway of children with UDT in our department from diagnosis to surgery, in order to assess our compliance with current guidelines and to identify any mismanagement in the process. This is the first study to examine the application of the cryptorchidism guidelines in Greece, with a view to improve the quality and the outcomes of the provided health services to the children.

## Materials and methods

A retrospective, observational analysis of males who underwent orchidopexy took place from January 2015 to April 2019, in a tertiary university hospital in Northern Greece. Three doctors used the hospital’s electronic medical files to retrospectively review the patient data for 51 months. They then recorded the age at cryptorchidism diagnosis, the patient age at orchidopexy, the meantime between diagnosis and surgery, and the laterality of the defect. The inclusion criteria were orchidopexy for congenital cryptorchidism in children and adolescents (0-16 years old) without any other related medical history. On the other hand, the patients who met one of the following criteria were excluded: genitourinary and neuromuscular anomalies, testicular torsion, and other clinical entities that may present cryptorchidism, such as acquired UDT, retractile testes, gliding testes, and postoperative cryptorchidism following operations applied on the inguinal area. After recording all the patients that met the inclusion criteria, we separated the sample into three groups named A, B, and C, based on the diagnosis age of UDT. More specifically, group A consisted of patients who were diagnosed with congenital cryptorchidism by the age of 0-11 months, group B included the males with UDT aged 12-18 months, and group C included the males with UDT diagnosis established at the age of >18 months. We also recorded the mean orchidopexy age and the meantime between diagnosis and surgery for each group. Primary outcomes were the age at diagnosis and the age at orchidopexy of males with UDT, while the secondary outcomes were the delay until surgery, as well as the comparison between groups.

Statistical analysis was performed using the Excel software (Microsoft Excel version 16.45 {2019}) (Microsoft® Corp., Redmond, WA) for data entry, and a descriptive statistical analysis of the sample’s data was completed. All the continuous variables had normal distribution and were presented as median values. They were compared using Student’s t-test. The p-values of <0.05 were considered significant for the statistical analysis.

## Results

In total, 217 children were diagnosed with UDT and received orchidopexy in our department. The majority of the patients (47.4%) had right-sided UDT, while 25.3% of them had UDT on both sides. For 80.2% of the patients, the UDT was the only condition diagnosed, while the rest (19.8%) had another clinical condition such as hypospadias, contralateral retractile testicle, and contralateral hernia/hydrocele. The characteristics of the patients are presented in Table 1.

**Table 1:** Characteristics of the sample patients. UDT: undescended testes

Patients’ characteristics	Total patients (Ν = 217)
0-11 months (group Α)	89 (41.0%)
12-18 months (group Β)	20 (9.2%)
>18 months (group C)	108 (49.7%)
UDT laterality	
Right-sided	103 (47.4%)
Left-sided	59 (27.2%)
Bilateral	55 (25.3%)
Only UDT	174 (80.2%)
UDT and other conditions (hypospadias, hernia, hydrocele, and others)	43 (19.8%)

There were 89 (41%) children diagnosed with UDT by the age of 0-11 months, and they constituted group A, while 20 males (9.2%) with diagnosis age at 12-18 months and 108 (49.8%) males with diagnosis age at >18 months constituted groups B and C, respectively (Table 2). The median age at diagnosis was 18 months (range: 1-164). When we calculated median age at diagnosis for groups A, B, and C separately, we found it to be five (range: 1-11), 15 (range: 12-18), and 71.5 (range: 19-164) months. The median age at orchidopexy was 23 months (range: 6-166), and for each aforementioned group, it was 11 (range: 6-82), 16.5 (range: 12-24), and 74 months (range: 20-166), respectively (Table 2).

**Table 2:** The primary and the secondary outcomes after reviewing males with UDT in our department. UDT: undescended testes

Primary/secondary outcomes	Group A (0-11 months)	Group B (12-18 months)	Group C (>18 months)	Total
Diagnosis age (months)	5 (1-11)	15 (12-18)	71.5 (19-164)	18 (1-164)
Orchidopexy age (months)	11 (6-82)	16.5 (12-24)	74 (20-166)	23 (6-166)
Delay until orchidopexy (days)	157 (1-692)	42 (1-183)	56 (1-635)	84 (1-692)

In total, the mean waiting time for orchidopexy was 84 days (range: 1-692), and for each group, it was 157 (range: 1-692), 42 (range: 1-183), and 56 days (range: 1-635) (Table 2). When comparing the waiting time between the diagnosis and the orchidopexy for the three groups, the children from group A had the longest delay until the scheduled surgery, which was also statistically longer than the delay in groups B and C (p _A versus B_ = 0.01 and p _A versus C_ < 0.0001). There was no statistically significant difference in the waiting time between group B (42 days) and group C (56 days) (p > 0.05).

## Discussion

The current study estimated the median age at diagnosis, the median age at orchidopexy, and the diagnosis to surgery time interval of children who received surgical treatment for UDT in one of the major and busiest pediatric surgery departments of Northern Greece. After trichotomizing the data based on the time of diagnosis into group A (0-11 months), group B (12-18 months), and group C (>18 months), only half of the patients appeared to have received a timely diagnosis and repair. More specifically, 109 patients (50.2%) (group A = 89 {81.65%} and group B = 20 {18.35%}) were diagnosed with UDT and were offered an orchidopexy until the age of 18 months, which is considered the upper age limit for timely treatment [5].

The current guidelines support the prompt surgical management of congenital cryptorchidism, in order to prevent the adverse events of untreated UDT [5,6]. In particular, Feyles et al. retrospectively investigated a cohort of young males with a history of UDT, and they described the associated long-term complications, which are the following: reduced fertility in adult life, the increased risk of testicular malignancy, the increased risk for testicular torsion, and the psychological effects due to cosmetic concerns and appearance in the person [7].

A meta-analysis by Kolon et al. followed and verified the protective role of early repair. They confirmed the necessity to perform orchidopexy by the 18th month of age (corrected gestational age), after estimating that for each following semester that the orchidopexy is delayed, a 6% increase in the testicular cancer risk and a 1% reduction in the fertility potential will occur in the adult life of the child [5].

Our data have highlighted the alarming finding that half of our patients (108 children {49.8%}) were referred, diagnosed, and operated after the age of 18 months, undoubtedly way above the recommended optimal times and a worrying finding whatsoever. Surprisingly, these findings are not uncommon in the literature. Although there has been a continuous, gradual decrease regarding the applied orchidopexy age from the 1950s until nowadays, as a result of the vast number of studies revealing the complications of the UDT and the advantages of the timely intervention, the age distribution of children undergoing orchidopexy remains higher and incompatible with the current guidelines [16,17]. There is no regional or national difference in the prevalence of such delays and the factors that lead to the delays worldwide. An extended analysis of selected American pediatric hospitals reported that only 43% of males with UDT had surgery by two years of age [18]. Similarly, a 10-year cohort study in Central Europe presented a poor implementation of the recommended orchidopexy time [19]. Α 11-year retrospective research in New Zealand also presented that only a small proportion of children with UDT are treated by 18 months of age [20], and accordingly alarming results were demonstrated by an observational study from Asia in 2014 [21]. There are two peaks of the applied orchidopexy in childhood, the one in the second year and the other in the 10th-11th year of the child’s life. Even if we exclude the conditions of acquired UDT, the surgical referrals and the performed orchidopexies still remain outside of the expected time frame [14].

The underlying causes of the broad orchidopexy distribution remain scarce. Many possible explanations have been proposed and could be summarized into four main categories: the doctor-related factors, the hospital policy, the patient and families, and the socioeconomic-related factors. In the first scenario, the long-term observation due to the lack of awareness and/or adherence to the guidelines expands the meantime between the diagnosis of UDT and final orchidopexy [17]. Recently, an observational study from South Europe highlights that most referrals for UDT are already beyond the recommended age [22]. Bašković et al. report that the lack of theoretical knowledge among pediatricians influences the surgery age [23]. Similarly, Gerber et al. blame the poor-quality clinical examination by the non-surgical referring physicians as an important factor in delayed surgery [24].

In the second scenario, a significant delay between referral, diagnosis establishment, and operation time was reported by Chen et al., due to the scheduled consultation appointments, the waiting list for surgery, and the hospitalization costs [21]. In our country, children are followed mostly by pediatricians and less by general practitioners. However, there is not an established hospital policy concerning the referral pathway protocol but rather a suggestion for pediatric surgery consultation to the parents. In addition, the patient’s characteristics, such as the medical history, demographic data, and medical insurance status, affect the surgery time [25]. A large retrospective study in Asia showed that males with comorbidities such as inguinal hernia, hydrocele, and urinary system diseases are more likely to receive a prompt repair, compared to those without these conditions (p < 0.001) [25]. The urbanization level of the patient’s residence affects the surgery time. A seven-year observational Balkan study of patients treated for UDT reported that males from urban areas were more likely to undergo orchidopexy during the recommended time period compared to males from rural areas [26]. Last but not least, the family’s economic, educational, and rational situation, as well as the general socioeconomic (in)stability, seem to influence the adherence to guidelines and the scheduled operation [21,22].

In our study, the diagnosis to surgery time interval was significantly longer for the group A patients who were diagnosed before the age of 11 months old compared to the group B and group C patients, while there was no difference between the group B and group C patients. This time interval reflects the response of the surgical team to delayed diagnosis. Both the group A and group B patients were operated in a timely manner, but since the group A patients had an earlier diagnosis, their time of operation was not actually delayed but followed the hospital operation scheduling policy. On the other hand, the group B and group C patients were operated rather quickly after diagnosis, in order either to complete treatment in a timely manner or to prevent the detrimental effects of cryptorchidism on the testis as soon as possible. This also underlines that hospital rules can be bent when there are scientifically established proofs, consequently leaving the delayed surgical referral as the only possible reason for delayed treatment.

Last but not least, the limitations of this study should be mentioned. The retrospective character of the study has a negative effect on the data quality. Also, the variability among the age of children at diagnosis (range: 1-164 months) and the age at final surgery (range: 6-166 months) was not adjusted in the statistical process. The following sample heterogeneity highlights that the interpretation of the results should be made with caution. However, we believe that these limitations are balanced by the carefully extracted patient data, the specific inclusion and exclusion criteria, and the defined statistical analysis.

Putting the pieces together, the management of males with UDT can be a reflection of health-care quality, including access, diagnosis, and optimal management by the health services [15]. Primary or congenital UDT is a very common condition that can be detected through routine pediatric screening, and then, the patient can be treated as an outpatient in a pediatric surgical department. In cases where UDT is even suspected, immediate surgical consultation and follow-up should be suggested. Some aspects of the management could be improved with educational updates among non-surgical health providers and parents. These updates could emphasize how to identify a potential undescended testicle, at what age, and the surgical referral time frame afterward. Thus, the increased guidelines’ awareness among primary health-care practitioners and parents could result in prompt diagnosis and treatment of males with UDT [27-29].

## Conclusions

In conclusion, half of the patients with UDT in our department were diagnosed and treated within the recommended time range. However, there is a remaining significant proportion of patients receiving delayed orchidopexy, and this finding points primarily to delayed referrals for pediatric surgery consultation. Thankfully, the more delayed the diagnosis, the quicker the operation, reflecting the prompt surgical team response and hospital rules’ flexibility. There is a need to increase the acknowledgment among parents, primary medical practitioners, and pediatricians about the timeline of UDT management, in order to decrease the threshold for timely reference and to increase compliance with the updated guidelines. At least for our country, the need for an established referral pathway has also been highlighted.
